# Ten principles for machine-actionable data management plans

**DOI:** 10.1371/journal.pcbi.1006750

**Published:** 2019-03-28

**Authors:** Tomasz Miksa, Stephanie Simms, Daniel Mietchen, Sarah Jones

**Affiliations:** 1 SBA Research & TU Wien, Vienna, Austria; 2 California Digital Library, University of California, Oakland, United States of America; 3 Data Science Institute, University of Virginia, Charlottesville, United States of America; 4 Digital Curation Centre, Glasgow, United Kingdom; Genome Quebec, CANADA

## Abstract

Data management plans (DMPs) are documents accompanying research proposals and project outputs. DMPs are created as free-form text and describe the data and tools employed in scientific investigations. They are often seen as an administrative exercise and not as an integral part of research practice.

There is now widespread recognition that the DMP can have more thematic, machine-actionable richness with added value for all stakeholders: researchers, funders, repository managers, research administrators, data librarians, and others. The research community is moving toward a shared goal of making DMPs machine-actionable to improve the experience for all involved by exchanging information across research tools and systems and embedding DMPs in existing workflows. This will enable parts of the DMP to be automatically generated and shared, thus reducing administrative burdens and improving the quality of information within a DMP.

This paper presents 10 principles to put machine-actionable DMPs (maDMPs) into practice and realize their benefits. The principles contain specific actions that various stakeholders are already undertaking or should undertake in order to work together across research communities to achieve the larger aims of the principles themselves. We describe existing initiatives to highlight how much progress has already been made toward achieving the goals of maDMPs as well as a call to action for those who wish to get involved.

## Introduction

Data management plans (DMPs) are documents accompanying research proposals. They describe the data that are used and produced during the course of research activities, where the data will be archived, which licenses and constraints apply, and to whom credit should be given. DMPs are awareness tools to help researchers manage their data and ensure that it will be of high quality, accessible, and reusable after the project has ended. DMPs are typically created manually, mostly by researchers using checklists and online questionnaires. They are required by funding bodies and institutions all over the world, e.g., the National Science Foundation (NSF) in the United States, the European Commission in Europe, and the National Research Foundation (NRF) in South Africa.

**Fig 1:**
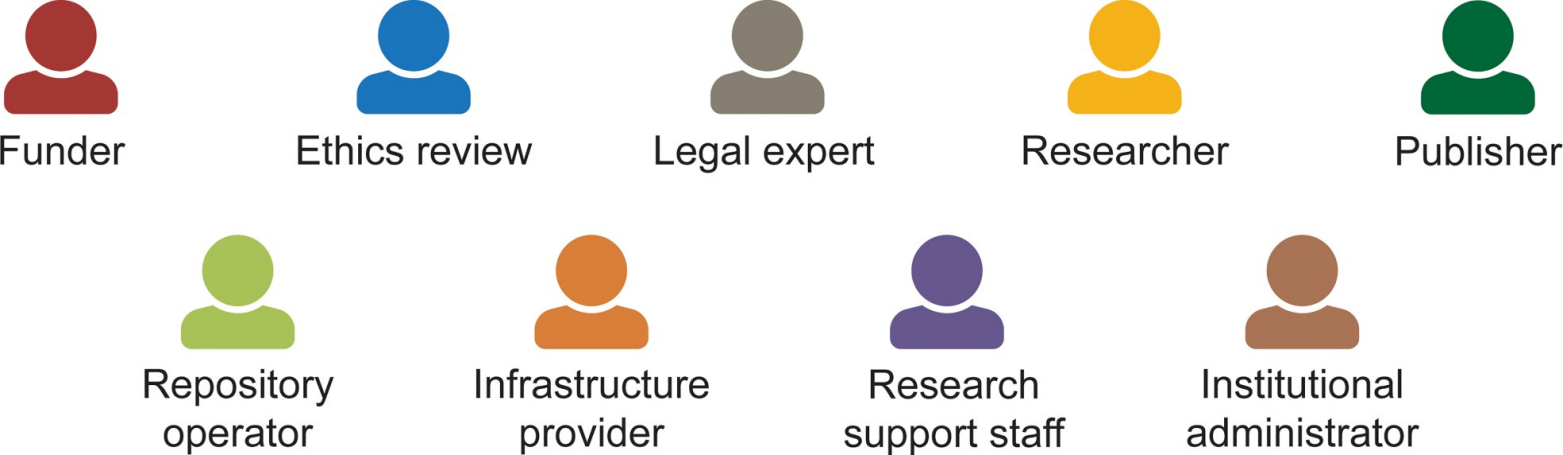
Target audience. Stakeholders with a role in realizing the maDMP vision. Funder: funding agencies and foundations that specify requirements for DMPs and monitor compliance. Ethics review: IRBs/REBs that authorize human subjects research. Legal expert: technology transfer offices; copyright and patent lawyers. Researcher: principal Investigator and collaborators, including postdoctoral researchers and graduate and undergraduate students. Publisher: purveyors of article and data publication services. Repository operator: general (e.g., Zenodo), disciplinary (e.g., GenBank, ICPSR), and institutional data repositories. Infrastructure provider: providers of systems for creating DMPs (DMPTool, DMPonline), grants administration, researcher profiles, etc. Research support staff: data managers/curators, research administrators, and data librarians. Institutional administrator: office of research/sponsored programs, chief information officers, university librarians, others. DMP, data management plan; ICPSR,; IRB, institutional review board; maDMP, machine-actionable DMP; REB, research ethics board.

The current manifestation of a DMP—a static document often created before a project begins—only contributes to the perception that DMPs are an annoying administrative exercise and do not support data management activities. Questions can remain unanswered, or the answers can be overly generic due to the use of free-form text. What DMPs really are, or at least should be, is an integral part of research practice, because today most research across all disciplines involves data, code, and other digital components (often in addition to physical materials, which can also be described in a DMP). A DMP describes digital research methods that will necessarily evolve over the course of a project; therefore, to be a useful tool for researchers and others, the content must be updated to capture the methods that are employed and the data that are produced. There is movement in this direction, e.g., Horizon2020 in Europe requires a DMP with varying levels of detail at different stages of a project, but this remains based on static text files. We continue to need a human-readable narrative, but there is now widespread recognition that the DMP could have more thematic, machine-actionable richness with added value for all stakeholders. This includes funders, repository managers, administrators, researchers, and so on (Fig 1)—in short, everyone who is part of the larger ecosystem in which data are produced, transformed, exchanged, and reused.

### What we propose

In this paper, we describe 10 principles for machine-actionable DMPs (maDMPs). The larger goal is to improve the experience for all involved by exchanging information across research tools and systems and embedding DMPs in existing workflows. This will enable parts of the DMP to be automatically generated and shared, e.g., with collaborators and funders. Furthermore, researchers whose data are reused in other experiments will gain recognition and credit because their data can be located, reused, and cited more easily.

To achieve this goal, all stakeholders must coordinate efforts to realize a new generation of maDMPs that contain an inventory of key information about a project and its outputs. The deployment of maDMP solutions can begin at a local level, e.g., within a research institution, country, etc. The basic framework requires common data models for exchanging information, as well as a shared ecosystem of services that send notifications and act on behalf of humans. Other essential components of the maDMP vision include machine-actionable policies, persistent identifiers (PIDs) used in new settings—e.g., Open Researcher and Conributor IDs (ORCIDs), funder IDs, and new initiatives such as Org IDs [1]—in addition to the removal of barriers for information sharing. By implementing and experimenting with these components, we believe that the global research community can reduce the administrative workload on all stakeholders and enhance the quality of recorded information.

For example, new and/or existing services could consume information provided by a researcher on the amount and type of data they will produce and automatically suggest a proper license, estimate costs of storage, and notify a repository operator to reserve space for a future data deposit. In this manner, we can reduce the input needed from researchers and make their decisions actionable, rather than just describing them.

Here is a list of potential benefits for each stakeholder (Fig 1):

- Funder: Structured information about who is producing the data (e.g., ORCIDs) and where data will be deposited (e.g., PID for repository listed in re3data.org) enables funders to monitor compliance through automated rather than manual processes.
- Ethics review: Relevant DMP content can be reused in institutional review board (IRB) or research ethics board (REB) applications. This provides important information about consent, etc. at the beginning of a project before data have been collected. It also provides a traceable record of IRB/REB approval to ensure research integrity.
- Legal expert: Relevant DMP content can be reused in patent applications. This provides important information at the beginning of a project to ensure that research is conducted in a manner that enables copyright and patent activities downstream.
- Researcher: Enables connections with experts throughout a research project for data management advice and support. Automated processes can facilitate DMP creation, enable others to update the DMP, streamline data preservation, and automate reporting. DMPs will also be an important source of information on experiment design and implementation.
- Publisher: Enables automatic generation of a data availability statement (from dataset digital object identifier [DOI]). Supports linking and proper citation of articles, datasets, and other outputs.
- Repository operator: Provides information about costs, licenses, metadata requirements, etc. up front. Enables capacity planning. Facilitates data ingest and preservation. Automated notifications at key points to update or verify information.
- Infrastructure provider: Information can flow between systems and does not have to be entered multiple times; it can be updated by appropriate stakeholders on behalf of researchers (which also improves quality of information) and aggregated for business intelligence.
- Research support staff: Can assess the quality of information contained in a DMP and offer feedback. Automated notifications at key points (e.g., grant awarded, data deposit, reporting) to provide support. Facilitates program development for consulting and support services.
- Institutional administrator: They can get a holistic view on the data used, processed, and created within the institution. This helps in better planning of resources needed to support data management infrastructure.

### What we do not propose

These 10 principles outline specific steps that must be taken to put maDMPs into practice and begin to realize their benefits. The principles are independent of any tool or technology and are not related to any specific DMP template or funding organization. We do not require implementation of all the principles by all global stakeholders simultaneously. The movement can proceed bottom-up from small-scale implementations that grow into a network of services. Finally, the principles do not contain guidance for researchers writing a traditional DMP, as those exist already and can be found in [2].

### Target audience

This paper is addressed to a wide range of stakeholders involved in research data management (RDM) workflows (Fig 1). The primary audience is those with the greatest ability to bring this maDMP vision to life, i.e., policy makers, funders, and institutions. Broad adoption by all stakeholders is required to achieve the benefits, but researchers cannot follow the principles if the infrastructure providers do not provide supporting systems.

### Methodology

We want to emphasize that maDMPs are part of a global community effort to improve traditional DMPs and the quality of research data (and metadata) more generally through automation while also reducing administrative overhead. The substance and inspiration for the principles is based on community-generated use cases from a workshop held at the International Digital Curation Conference (IDCC) in Edinburgh in 2017 that gathered almost 50 participants from Africa, America, Australia, and Europe [3]. The 10 principles themselves have gone through multiple drafts since then via consultations with Research Data Alliance (RDA) and FORCE11 groups focused on DMPs. The current phrasing takes into account all of the feedback received through various channels in the RDM community: all of the stakeholders represented in Fig 1 have participated in the events described above and provided input as users of our DMP services (e.g., DMPTool, DMPonline).

### How to read the principles

**Fig 2:**
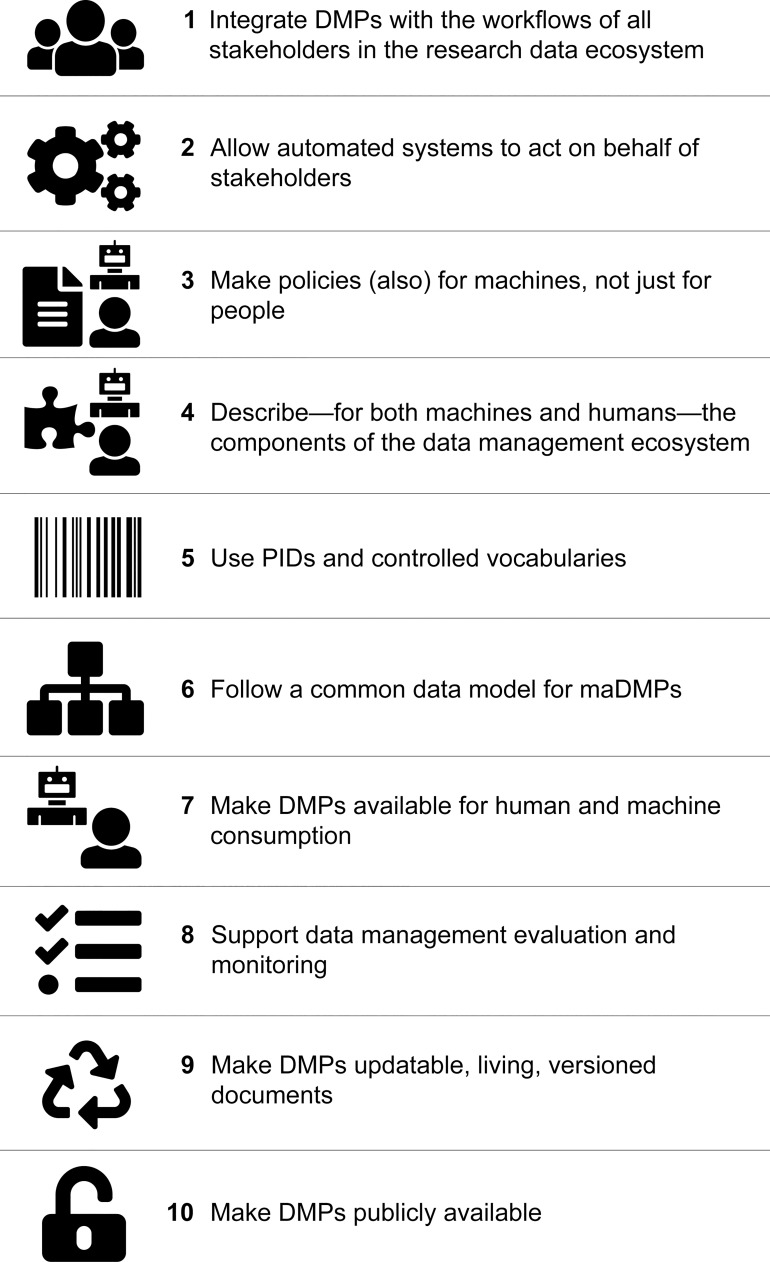
Ten principles for maDMPs at a glance. DMP, data management plan; maDMP, machine-actionable data management plan; PID, persistent identifier.

All 10 principles are equally important and can be read in any order (Fig 2). Some principles depend on others, e.g., to implement a common data model, we need PIDs and controlled vocabularies (i.e., Principle 6 depends on Principle 5). We indicate these dependencies and relationships between principles in the text.

The principles also vary in scope and specificity. Some are narrower (e.g., Principle 3: Make policies [also] for machines, not just for people), and some are broader (e.g., Principle 8: Support data management evaluation and monitoring). This is because principles address a combination of technical, organizational, and social issues that can be defined on different levels of granularity.

Another important point is that we consider data and metadata jointly throughout the paper. This encompasses basic project metadata that should be part of any DMP (e.g., project title, abstract, institution, names of the people involved, and associated identifiers, as per Principle 5) as well as the research data that are described in the DMP and accompanied by appropriate metadata when preserved in a repository. It also extends to things like metadata about the repository and related policies. The idea is to apply these principles to any piece of information or infrastructure that supports effective and efficient management of research data.

The principles can be understood in a different manner by different stakeholders within the DMP ecosystem. When developing the principles, we kept in mind three roles that represent a majority of stakeholders: (1) policy making and infrastructure provision, (2) DMP authoring and updating, and (3) using and reusing (DMPs directly, data indirectly through DMPs).

Where it is appropriate to do so we distinguish the principles by stakeholders, but readers should note that many roles and responsibilities overlap and vary across domains, institutions, countries, and projects as well as along the timeline of a research project.

### How to get involved

You can begin implementing maDMPs on your own, as services and systems should be customized for your needs. Join the RDA Working Groups to contribute to their activities to share ideas and avoid duplication of effort. Consult the list of projects on https://activedmps.org/ and connect with others working in this area.

## Principle 1: Integrate DMPs with the workflows of all stakeholders in the research data ecosystem

(This principle applies to all stakeholders [Fig 1].) Good data management requires precise information on various aspects of data ranging from methodological and technical details on formats and infrastructure to legal and ethical aspects of data collection and reuse.

Authoring DMPs should not be the responsibility of a single person but has to become a collaborative exercise, in which various stakeholders who are knowledgeable in their domains and adjacent parts of the data management ecosystem share their expertise. Only then can we ensure that the right information is provided and can be acted on by others.

**Fig 3:**
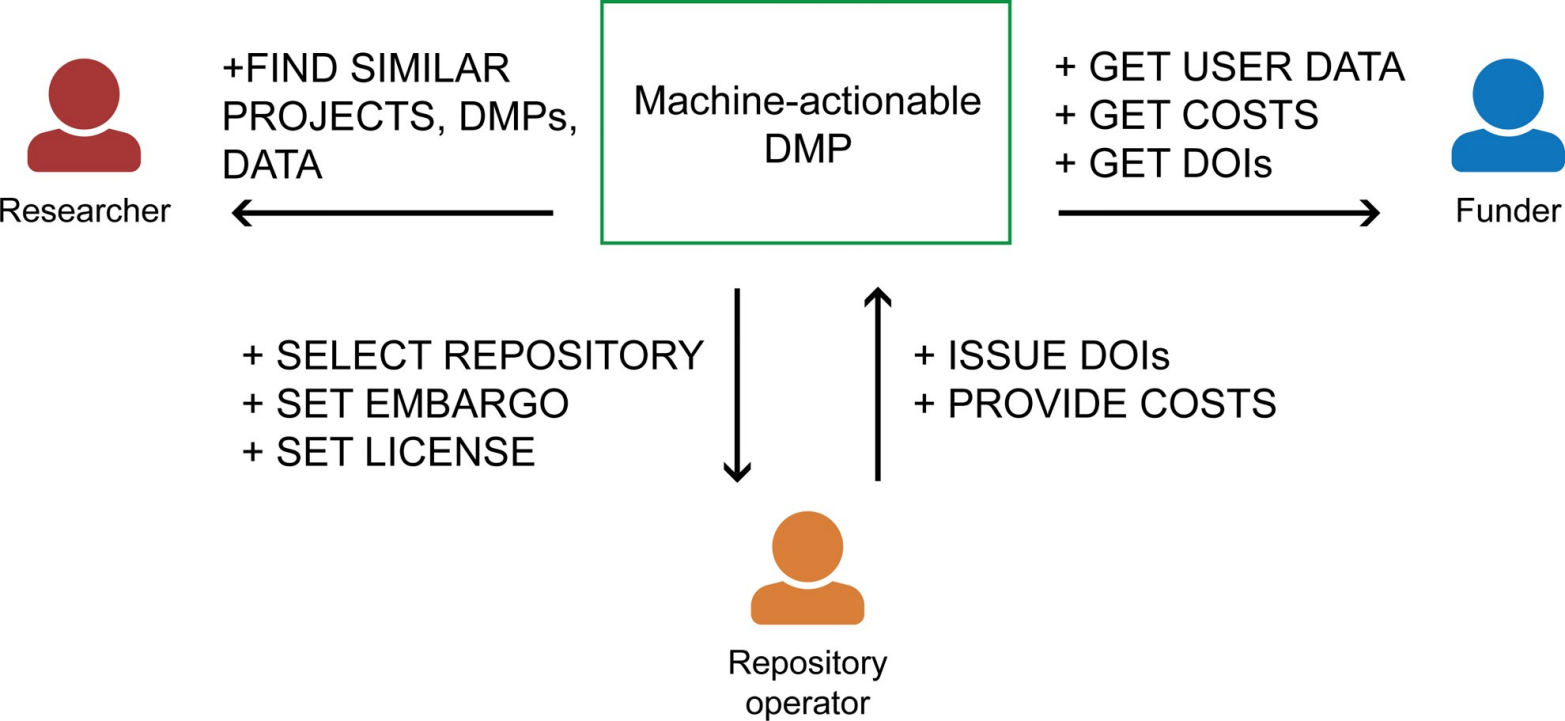
Stakeholder interactions. Examples of stakeholder interactions within the ecosystem of maDMPs. Stakeholders communicate with each other by exchanging information through DMPs. For example, a repository operator can select a proper repository, set an embargo period, and assign a correct license to data submitted by researchers. In return, a system acting on behalf of a repository operator provides a list of DOIs assigned to the data and provides information on costs of storage and preservation. This in turn can be accessed by a funder to check how the DMP was implemented. Researchers can browse DMP catalogues using a variety of filters that allows them to discover projects using similar methodologies or infrastructure or producing similar outputs. DMP, data management plan; DOI, digital object identifier; maDMP, machine-actionable DMP.

Information provided in DMPs is also consumed by multiple stakeholders (Fig 3). For example, repository operators set embargo periods and assign licenses for repository content based on information in the DMPs that was provided by researchers, while research funders check whether research outputs that have been published or deposited in repositories follow relevant policies and guidelines, such as the principles findable, accessile, interoperatble, and resuable (FAIR) [14].

Multiple stakeholders provide information in DMPs, and multiple stakeholders consume it, so coordination among them is key. Traditional DMPs are typically written at the beginning of a project and rarely used later. As a result, opportunities to use, update, and reuse the information held within them are missed. Moreover, the many-to-many relationships of a wide variety of stakeholders contributing and/or consuming different elements of DMPs are not currently supported by DMP-related infrastructure. maDMPs will formalize workflows that truly engage the appropriate stakeholders at the appropriate stages of a research project. To change this, we need to involve all stakeholders throughout the data management lifecycle, starting from project planning, through project execution, to project end and preservation (cf. Principle 9). The maDMPs and their common model (cf. Principle 6) will facilitate the structuring of information, but this has to be complemented by organizational and technical means that involve the various stakeholders at all stages of data management who provide and reuse information from DMPs.

Organizational changes should ensure that tasks related to data management become routine and not ad hoc actions. For example, legal experts should be involved in selecting licenses, while information technology (IT) experts should advise on the best tools and infrastructure to manage data. This has to be supported by technical means that allow systems to automatically act on behalf of stakeholders (cf. Principle 2), e.g., by sending automatic notifications to specific stakeholders when input or other actions are expected from them. This will not increase the workload because many requests should involve routine data management tasks that can be handled in an automated manner, e.g., a university recommends a certain license for sharing data. Other nonstandard requests can be processed in an organized way, replacing what are currently ad hoc processes. In [13], authors describe results of a stakeholder consultation that collected information on how needs for information of particular stakeholders evolve over phases of the research data lifecycle with respect to maDMPs.

## Principle 2: Allow automated systems to act on behalf of stakeholders

(This principle applies to any stakeholder who manages information in DMP-related systems [Fig 1]—repository operator, infrastructure provider, institutional administrator, ethics review, legal expert, and publisher.) The full involvement of all stakeholders in RDM (cf. Principle 1) depends on having systems to automatically act on their behalf, thus reducing the need for human interaction while helping to focus the remaining human interactions on tasks that cannot be automated readily.

Some of the information captured in a DMP is already available electronically, so instead of entering it again, it would be helpful if the relevant bits could be fetched from appropriate sources, perhaps after consistency checks with other sources for quality assurance.

To make this happen, we need to integrate systems and allow stakeholders to expose services that automate tasks and act on their behalf, for example:

- Collating administrative data: a service that acts on behalf of researchers or other DMP authors and collects administrative information, such as affiliation, grant number, and postal or email addresses from institutional databases like Current Research Information Systems (CRIS) or Research Information Management (RIM) systems to prefill the DMP. Information could also flow from the DMP into the CRIS, and if previous DMPs are in the system, relevant bits (e.g., about instrumentation or data formats) could be fetched to assist in authoring or reviewing another DMP.
- Cost estimation: a service that acts on behalf of repository operators and implements a cost model of a repository to provide automatic estimates of costs of storage and preservation based on input parameters such as amount of data, type of data, project duration, etc. There is research on cost models and ways of comparing them [16], but there is still no such service in place.
- License selection: a service that acts on behalf of legal experts and proposes a license for data sharing, taking into account policies that apply to the project and type of data. For example, if the institutional policy recommends open access publishing and the data do not contain sensitive information, then CC0 could be the default setting for data, and CC BY for text and media. There is already a wizard from EUDAT [4] that offers similar functionality.
- Storage booking: a service that acts on behalf of an repository operator and reserves storage space for the duration of a project if a repository suitable for the expected types and amounts of data and meeting relevant policy requirements can be found. Furthermore, such a service can help repository managers plan infrastructure investments when they know how much new data is expected in advance.
- Data deposit: a service that acts on behalf of a repository operator to deposit data and associated metadata, using information from the DMP such as embargo periods, license types, and metadata standards, to automatically set properties of ingested data.
- Validation and compliance: a service that acts on behalf of a funder and checks compliance with its policies, e.g., by checking whether data described in a DMP is accessible by the indicated time and under appropriate licenses.

These examples show that automation is possible for the majority of stakeholders during various phases of a project lifecycle. This helps to save time and reduce costs while also providing more precise information.

Apart from services automating tasks, we need a system that triggers automated notifications when human intervention is needed (cf. Principle 7). For example, it can create a ticket and assign a human who will then either provide the missing information directly or contact the researcher if clarification is needed.

## Principle 3: Make policies (also) for machines, not just for people

(This principle applies to all stakeholders who provide data-related policies [Fig 1]: funder, repository operator, infrastructure provider, institutional administrator, ethics review, legal expert, and publisher.) Interactions among humans as well as between humans and human-made systems are guided by cultural norms, some of which are formalized as legal documents like guidelines, contracts, policies, or laws. For simplicity, we refer to them collectively as policies.

There may be various policies relevant to a given DMP, e.g., on data sharing, data quality, data security, or ethical review. While policies usually agree on a broader goal, they often handle details in different fashions, which makes it hard for any of the relevant stakeholders to find out whether data are compliant with applicable policies.

Policy statements may be very broad, e.g., “Research data will be managed to the highest standards throughout the research data lifecycle as part of the University’s commitment to research excellence” [5], or they may be specific enough to be easily applied and tested. More specific requirements could be broken down into a set of principles checking certain properties (e.g., Is the resource available? Does it have a PID? Is it registered?).

Data policies should themselves be machine actionable, at least at some basic level, to assist in the evaluation of data management practice. This can be achieved in several ways, e.g., by

- composing policies using machine-actionable policy elements (cf. [6]),
- including a machine-actionable section into policy documents, or
- complementing a policy with an associated machine-actionable document (e.g., an appendix).

The common feature of these three approaches is that the key requirements of the policy should be expressed in a format that machines can act on, i.e., using a consistent predefined structure and a controlled vocabulary. While humans might reasonably object to following policies, machines are happy to comply when properly instructed. Investing effort in making data policies less ambiguous, more discoverable, and machine-actionable will pay dividends, helping funders, publishers, and other stakeholders achieve much higher adoption. An example of machine-actionable policy was developed by the PERICLES project funded by the European Union and is used in the domain of digital preservation ([7]).

## Principle 4: Describe—For both machines and humans—The components of the data management ecosystem

(This principle applies to all stakeholders who provide DMP-related systems [Fig 1]: funder, repository operator, infrastructure provider, institutional administrator, ethics review, legal expert, and publisher.) A common problem faced by researchers is how to find a suitable repository for data sharing and preservation. There is a wide range of repositories that differ in the types and amounts of content they accept, levels of trust, geographical location, costs, licensing, and so on. Each repository provides this information in a different form or even language—sometimes, it is included in the terms of use, in other cases it is part of a frequently asked question (FAQ), or it may not be specified at all and only provided upon request.

If we provide a common way to describe specific components of a data management ecosystem—such as repositories—then these components can be readily discovered by humans and machines. Specifically, in the case of machines, we would be able to create services (cf. principle 2) that can suggest a repository using information already provided in a DMP. Thus, authors of DMPs would be presented with a list of repositories that fulfill their criteria, and the selection will be narrowed down to those that are relevant.

Conversely, stakeholders who described their services and infrastructure using such standard terms could be informed of parties who selected their services in a DMP, and have greater confidence that those parties are aware of the associated conditions. In the case of repositories, such conditions could be matching data and metadata standards, and checking such matches reduces the effort required for ingesting and maintaining the data.

This principle goes beyond repositories to include all other components of the data management ecosystem that need to be discovered by humans and machines. It should not be confused with Principle 6, which recognizes the need for a common data model for DMPs themselves, because the common way to describe specific components of the data management ecosystem enables service discovery (i.e., finding resources that may be relevant for DMP creation or automated notifications), while a common data model for DMPs is a way to model information that is, at least in principle, known to the DMP authors.

This principle is not about starting from scratch, but rather leveraging the considerable amount of information and functioning services already in existence, some of which already provide the necessary application programming interfaces (APIs) to support maDMPs. Two different registries—re3data[8] and OpenDOAR [9]—contain critical information about thousands of data repositories (e.g., content types, location, preservation policy, etc.). Each registry is curated manually, and each repository must undergo a review before being added to the list. Re3data provides everything in an openly accessible, machine-actionable format through its API and is currently working on a recommender service for the earth and space science domains as part of the American Geophysical Union Enabling FAIR data project. A related project called Science Europe Domain Data Protocols [10] is a proof of concept that aims to define standardized, machine-actionable building blocks for DMPs based on domain-specific protocols for data management. Similar concepts exist for open data (e.g., Tim Berners-Lee's 5-star open data [11]). We cannot convert all PDFs into linked data, but this is a vision we should pursue if we want data to be machine actionable.

## Principle 5: Use PIDs and controlled vocabularies

(This principle applies to all stakeholders [Fig 1].) To make DMPs explicit and understandable for all stakeholders (cf. Principle 1 and Principle 7), we need well-defined terms and precise identification of resources.

The free-form text fields dominating traditional DMPs can contain complex and/or ambiguous terms. This can lead to situations in which it is not clear what data were used in an experiment, where the data will be deposited, or to whom the provisions in the DMP apply.

Sometimes, the opposite is the case: the wording is specific and thus understandable in a very narrow context, requiring implicit knowledge on the part of reusing parties. This can become an issue when data are reused in a different domain or even when the DMP is co-created by various stakeholders (cf. Principle 1).

Furthermore, DMPs are living documents (cf. Principle 9), and the amount and granularity of information contained within them evolves over time—from high-level estimates and expectations down to precise descriptions of actions that have actually been taken.

For this reason, to implement maDMPs, we need to use controlled vocabularies and PIDs whenever possible. Controlled vocabularies provide a list of common, well-defined terms that can be used to annotate data or to provide users with a limited list of options to choose from when describing their data or associated workflows. PIDs provide a way to identify and locate resources. They can be used to refer to people and publications, as well as datasets, file types, repositories, organizations, policies, and other elements of the research data ecosystem. For example, principle investigators can be identified using their ORCIDs, and their data using DOIs. Additional PID systems already exist and/or can be developed to identify other resources, such as specific instances of a given repository software, scientific protocols (e.g., https://www.protocols.io/), or a cell line (e.g., https://scicrunch.org).

In cases in which an identification system does not exist, maDMPs can employ controlled vocabularies instead. For example, researchers should be able to choose their affiliation by default from a controlled list of institutions. In a similar fashion, they should be able to select rather than type the appropriate metadata standard or a license for their data. This would alleviate generic and meaningless descriptions commonly found in traditional DMPs, such as “best community practices and standards will be used to document all outputs produced by researchers working on this project.”

## Principle 6: Follow a common data model for maDMPs

(This principle applies to all stakeholders who provide DMP-related systems [Fig 1]: funder, repository operator, infrastructure provider, institutional administrator, ethics review, legal expert, and publisher.) A common data model is a medium for exchange of information between stakeholders (cf. Principle 1). It provides information in a machine-actionable form, thus enabling interoperability of tools and services that act on behalf of stakeholders (cf. Principle 2).

The common data model is not a prescriptive template or a questionnaire but provides a reusable way of representing machine-actionable information in a structured way on themes covered by DMPs. It models information, which contrasts with the free-text information gathered by the questionnaires known from traditional DMP tools.

Due to a wide range of topics covered by the DMPs used in different disciplinary, national, or other contexts, the model should be modular. It should have a core model common for all DMPs and a clear mechanism for including extensions that describe specific aspects of data management or that address specific domain requirements. It should also reuse existing standards, controlled vocabularies, and models to organize information in a systematic way (cf. Principle 5).

The common data model does not affect the internal architecture of specific components within the data management ecosystem—each component (e.g., a repository) can model the information internally in the way that is best for its purpose, but when information is exchanged across components, then this information must be modeled using the common data model.

The common data model remains transparent to the stakeholders authoring and updating DMPs: when their input is needed, they will be notified and presented with relevant information (cf. Principle 2). The common data model is used by these tools to read and write information to and from the maDMP and to automatically take actions based on the information therein. A common data model is currently under development in the context of the RDA DMP Common Standards Working Group, with an estimated delivery date in early 2019 and widespread intentions for community adoption. The first author is a co-chair of the group.

## Principle 7: Make DMPs available for human and machine consumption

(This principle applies to all stakeholders [Fig 1].) The intended audience for traditional DMPs includes the proposing researchers, reviewers, and the funder (e.g., program officers) at the grant proposal stage. However, in practice anecdotal evidence from review panels and conversations with funders suggests that DMPs are not routinely evaluated as part of grant proposals and no funders have published review criteria. At best, they will be read only a few times by a human.

By converting DMPs into living documents (cf. Principle 9), they become more likely to be consulted multiple times throughout the course of research. This works best if not just the most current version is readily accessible but differences between versions can be assessed by both humans and machines.

It would also be helpful if interested parties could subscribe to automated notifications of changes to a specific DMP, ideally in a way that allows for different levels of granularity. For instance, project collaborators may be interested in the full content of the DMP, whereas the repository named as the destination of a specific subset of the data may only be interested in changes to the amount, licensing, deposition date, or format of that specific data subset. By the same token, repository operators should also receive automated notifications about a canceled booking for a project that has been rejected or no longer intends to deposit data there.

Enabling such granular notifications requires the DMP to be machine actionable at corresponding levels of granularity. This necessitates avoiding free text and providing structured information whenever possible. Some form of human-readable narrative will remain necessary but DMP content that is structured, machine readable, and actionable increases the potential for reuse.

By turning DMPs into public documents (cf. Principle 10), they are more likely to be consulted by multiple humans and machines. Having maDMPs would also facilitate the aggregation of DMPs at the available levels of granularity. For instance, infrastructure providers or funders may be interested in dashboards aggregating project-based DMPs on an ongoing basis and reslicing them in various ways, e.g., by the institutions associated with these DMPs, by the designated infrastructure, by the funding mechanism, or by the kinds of data. The successful implementation of this principle requires that DMPs no longer be treated as closed grant materials by funders, researchers, and institutional administrators. Alternatively, the RDA Exposing DMPs Working Group plans to provide recommendations about what subset of information contained in a DMP should be made open (e.g., project details but perhaps not the full content) and/or what kind of mediated access should be enabled.

Finally, there will be still questions that can only be answered by humans, e.g., about ethical issues [15]. In such cases, an informed guess can cause more problems than solve. Human input is inevitable. For this reason, maDMPs cannot be an invisible virtual entity living in a closed information and communications technology (ICT) infrastructure but must be a piece of information that can be edited by a human.

## Principle 8: Support data management evaluation and monitoring

(This principle applies to all stakeholders [Fig 1].) Despite our emphasis on improving the quality of DMPs to enable researchers to manage their data, we acknowledge that funders and policy makers drive the demand for DMPs. For this reason, the structure of DMPs and ecosystem of services must support compliance monitoring. If DMPs are to be taken seriously, they must be evaluated along with grant proposals and during active stages of research. Reviewers and other stakeholders still need a human-readable narrative, but providing policies in machine-actionable formats (cf. Principle 3) would also assist in automated monitoring, e.g., of research outputs or compliance with applicable policies. DMPs should be explicit about the policies they are meant to comply with, and include version numbers and PIDs to avoid ambiguity.

Involving stakeholders in the process of DMP authoring (cf. Principle 1) and use of controlled vocabularies, PIDs (cf. Principle 5), and a common data model (cf. Principle 6) improves the quality of information contained in DMPs. This is because fine-grained information will be provided in a structured way and many associated tasks can be automated.

For example, in an early phase of a DMP creation, the tools can check whether a selected license for data sharing is compliant with a funder policy. In a later phase, when data are created and are supposed to be deposited in a repository, the tools can automatically check whether the data in question were deposited there and were accessible and licensed as prescribed by applicable policy. This would enable relevant stakeholders, especially grant reviewers and funders, to monitor DMP compliance through automated processes.

However, maDMPs should never be an evaluation means on their own. DMPs must reflect reality (or realistic planning), even if that differs from best data management practices. DMPs also cannot impose limits on research methodology and must permit investigations to be conducted using any technology of choice.

## Principle 9: Make DMPs updatable, living, versioned documents

(This principle applies to all stakeholders [Fig 1].) It is unhelpful to think of DMPs as static documents. They should not just be seen as a “plan” but as updatable, versioned documents representing and recording the actual state of data management as the project unfolds. The notion of Data Management Records [12] to move beyond a plan has been put forward in this vein. The act of planning is far more important than the plan itself, and to derive value for researchers and other stakeholders, the plan needs to evolve. DMPs should track the course of research activities from planning to sharing and preserving outputs, recording key events over the course of a project to become an evolving record of activities related to the implementation of the plan.

Changes to maDMPs should trigger notifications at configurable levels of granularity to inform interested stakeholders accordingly (cf. Principle 7). For example, such notifications could inform research communities about amendments to the conditions under which forthcoming datasets will be made available or alert them as the datasets are deposited. As well as issuing notifications, systems could exchange updated data directly. As a new event is recorded in one system, it could automatically pass the new entry to CRIS/RIM platforms, grant management systems, repositories, or other related tools.

Updating the DMP might not always need human intervention. Some of the changes could be done automatically, triggered by events elsewhere in the research ecosystem, e.g., when data are deposited, the DMP could be updated with the timestamp and PID of the dataset. Conversely, some of the changes to a DMP (e.g., personnel changes) may need to be made by hand but could trigger notifications elsewhere in the system. In both cases, this requires that the information is machine actionable and that the notification mechanism is linked to some tracking tool that is aware of the relationships of the given DMP with relevant external resources and actors.

## Principle 10: Make DMPs publicly available

(This principle applies to all stakeholders [Fig 1].) The DMP is the earliest concrete indication of what data will be created in the framework of a research project and how it will be managed. Sharing and co-creating the DMP within the project team during the ideation and planning stages helps to specify the research methodology, to estimate required resources, and to produce a plausible timeline for data release.

Sharing it beyond the project team—e.g., within an institution, with repositories, funders or ethical review boards—from early on (as per Principle 1) helps streamline data-centric interactions between the various stakeholders over the course of the project.

Stakeholders with access to multiple DMPs (or consistent sections thereof) can aggregate them and—particularly for the subset that is machine actionable—mine the information contained therein and reslice it by the different parameters of the DMP data model (cf. Principle 6 and Principle 7). This informs RDM service delivery, facilitates monitoring and evaluation (cf. Principle 8), and stimulates the development of tools to explore such DMP corpora and to enable humans and machines to interact with them (cf. Principle 2 and Principle 7).

Ideally, DMPs should be shared early and often (cf. Principle 9) throughout the research process and as broadly as possible. When this is not feasible, they could be shared with a delay (e.g., at project end) or in limited contexts (e.g., within an institution) or in part (e.g., project metadata such as grant number, abstract, related outputs). The reasons for not sharing earlier, in full, or more broadly should be stated in a machine-actionable manner, e.g., through a standardized template in which the opt-out is justified using a controlled vocabulary. This would allow stakeholders to gather data about such circumstances and could inform future data management policies.

If maDMPs are shared in public and under an open license, anyone can aggregate them, reslice the corpora, use, and re-share the resulting information. Such front-ends to maDMP collections could be generic—which would help with the standardization and spread of good data management practices across domains—or be tailored for specific audiences, e.g., to facilitate discovery in a given area or education about research in the domain, including associated data management practices.

Another important use case for sharing DMPs in public is to accompany data that are described by the DMP and deposited in a repository. Because different sets of data may differ in parameters like their thematic scope, their file types, size, or sharing restrictions, they are often not shared in the same way, and it is hard to get an overview of what data have been shared by a given project. If each dataset or other research output—irrespective of where it was deposited—would always point to the appropriate version (cf. Principle 9) of the DMP in a machine-actionable manner, users who discover any part of that project's output could easily use the DMP to find the other parts.

This way, individual DMPs would act as a hub to project-level research outputs, and aggregations of DMPs as hubs to research more generally, including to planned or ongoing research and to research infrastructure.

While making an individual DMP machine actionable or versioned or public is beneficial in terms of data management and discovery, the real benefits come once many DMPs are machine actionable and versioned and public.
